# Novel metal based nanocomposite for rapid and efficient removal of lead from contaminated wastewater sorption kinetics, thermodynamics and mechanisms

**DOI:** 10.1038/s41598-022-12485-x

**Published:** 2022-05-19

**Authors:** Elsayed A. Elkhatib, Mohamed L. Moharem, Ahmed F. Saad, Farida A. Attia

**Affiliations:** 1 Departments of Soil and Water Sciences, College of Agriculture (Elshatby), Alexandria University, Alexandria, 21545 Egypt; 2 Regional Center for Food and Feed, Agricultural Research Center, Alexandria, Egypt

**Keywords:** Environmental sciences, Solid Earth sciences, Chemistry

## Abstract

A sol–gel method was utilized to prepare a novel nanocomposite adsorbent (nMgO/bentonite) and was tested for Pb(II) removal from aqueous solutions. The produced nanocomposite was investigated using, SEM–EDX, XRD, and FTIR analyses before and after Pb adsorption. Adsorption equilibrium and kinetic experiments were run in batch system under different conditions of pH, adsorbent dose, competitive cations, contact time and temperature. The results exhibited rapid Pb(II) adsorption by the nanocomposite in the first five min. Experimental lead adsorption equilibrium and kinetics data fitted well to Langmuir and power function models, respectively as indicated from the lowest standard error (SE) values. The calculated Langmuir maximum adsorption capacity (q_max_) value of nanocomposite (75 mg g^−1^) was 4.5 times higher than that of bentonite (16.66 mg g^−1^). Moreover, the highest quantity of Pb(II) uptake was achieved at temperature of 307 K and pH 9. The Langmuir sorption capacity of the nanocomposite for Pb(II) increased from 75 to 145 mg g^−1^ with increasing temperature from 287 to 307 K. The thermodynamic parameters of Pb(II) adsorption by the nanocomposite affirm the spontaneous and endothermic nature of the adsorption process. Lead adsorption mechanisms by the nanocomposite were proposed and discussed.

## Introduction

Increasing heavy metals concentration in various environmental systems (soil, water, and air) are mainly related to industrial activity enlargement all over the world and could transfer to food chain causing serious impacts on environment and human health^1,2^. Lead is one of the highly toxic heavy metals at low concentration and could have a detrimental effect on biological systems generating multiple diseases and disorders^3^. Thus, there is a growing demand for eliminating the toxic lead from the polluted areas such as water stream and wastewater to avert its hazardous impacts^4^.

Several techniques have been commonly applied for Pb(II) removal from contaminated water encompasses ion-exchange^5^, membrane filtration^6^, electrolysis^7^, coagulation^8^, flotation^9^. However, most of these techniques are expensive and need high concentration of heavy metals for efficient removal^10^. On contrast, adsorption-dependent technique considers a promising approach in heavy metals removal field as a results of its low operational cost, design simplicity, rapid operation, and high efficacy even at low pollutant concentrations^11,12^. Different materials have been used as heavy metals adsorbents such as mineral, organic or biological source, zeolite, bentonite, agricultural waste, industrial byproducts, and polymeric materials^13,14^. Yet, great efforts have been occurred to increase the surface activity and capacity of such adsorbents towards heavy metals^15–18^.

Bentonite clay has been commonly used for elimination of diverse contaminants from water due to the abundance of adsorption sites available on its surface towards heavy metals as well as its eco-friendly and green economic nature^18^. However, bentonite clays, in their natural state, possess low adsorption capacity and for that reason numerous attempts have been performed to enhance their capabilities in removal of organic and inorganic contaminants from wastewaters using different techniques^13,18^.

Nano-sized materials possess large surface area to volume ratio^19–21^ and various nano-materials have been successfully used in wastewater treatment for potential decontamination of industrial effluents, surface water, ground water, and drinking water^22–26^. Because nano-sized metal oxides such as nano-magnesium oxides (nMOs) exhibit high adsorptive efficiency for toxic metals from aqueous solution^27^. We hypothesized that modification of bentonite surface by coating with Mg oxides nanoparticles as a new technique may greatly enhance the reactivity and the adsorption affinity of bentonite for Pb(II) ions and could be a potential solution for improving the efficient use of bentonite clays in Pb(II) removal from contaminated water.

To the best of our knowledge, the present study was designed to synthesize the novel nano-composite (nMgO/bentonite)—*for the first time*—to enhance the sorption capability of bentonite for Pb(II) removal from contaminated water. The specific objectives of this study were to (i) produce nMgO-Bentonite nano-composite and characterizes its surface morphology and chemical composition using SEM–EDX, XRD and FTIR analyses, (ii) determine Pb(II) adsorption capacity under optimal conditions of pH, adsorbent dose, competitive cations, contact time and temperature, (iii) examine the reutilizing prospect of the nanocomposite, and (iv) evaluate the efficiency of the produced nano-composite for Pb(II) removal from industrial wastewater.

## Materials and methods

### Preparation of (nMgO-bentonite) composite

Sol–gel method was utilized to synthesize magnesium oxide nanoparticles^28^. Magnesium acetate tetra hydrate [Mg(C_2_H_3_O_2_)_24_H_2_O] and ammonia solution was used as the premier precursor and precipitating tool, respectively. Typically, 0.1 mol L^−1^ of the initial precursor was prepared in distilled water (200 mL) and then was stirred for 10 h using magnetic stirrer. Hereafter, 15 mL of the precipitating tool was added to the solution under certain conditions of temperature (80 °C), pH (8), and 16 h continuous stirring using temperature controlled orbital shaker to get the white Mg(OH)_2_ precipitate. The precipitate was then dried at 120 °C for 3 h and crushed using mortar pestle. Lastly, the fine powder was superheated at 600 °C for 6 h and then shriveled again to get the white powder of magnesium oxide. Bentonite clay was gained from Egyptian Nano-technologies Company at Borg El Arab city and sieved using 53 µm sieve. The bentonite composition of is presented in Table S1 (Supplementary Materials). The nano-composite (nMgO-Bentonite) was synthesized by adding nMgO to aqueous suspension of bentonite and stirred for 24 h. The resulting solid phase was detached by pouring, washed repeatedly with distilled water, and then dried at 105 °C for 24 h.

### Characterization

Morphological description and elemental analysis of bentonite, and (nMgO-bentonite) nano-composite were determined by using SEM supplied with ED spectroscopy (SEM–EDX) (INCAx-Sight, Oxford Instruments, UK). The crystalline nature of the three adsorbents were analyzed using XR Diffractometer (Bruker AXS D8 Advance). The specific surface area determination was performed using surface area analyzer (Quantachrome, USA). The nanoparticle size distribution of nanocomposite (nMgO-Bentonite) was measured using a Zetasizer Nano ZS (Malvern Panalytical) and is shown in Fig. S3. Adsorbent surfaces-functional groups were investigated using Fourier transform infrared spectroscopy (FTIR).

### Adsorption kinetics

Kinetic adsorption experiments were performed in 50 mL polyethylene centrifuge tubes; 0.2 g of bentonite or (nMgO-bentonite) nano-composite were added to 500 ppm Pb(II) solution as Pb(II) (CH_3_COO)_2_ and agitated for time periods (5 min–24 h) at room temperature (25 ± 2 °C). The mixtures were centrifuged for 10 min at 4000 rpm and then filtered through a 0.45 μm Millipore filter. Atomic absorption spectrometer (contrAA 300) was used for measuring Pb(II) concentration in the supernatant solutions. Four kinetic adsorption models were evaluated for their suitability for the sorption data.

### Adsorption isotherms

Lead adsorption equilibrium studies were run in batch system at room temperature (25 ± 2 °C) using 0.2 g of each adsorbent and lead acetate at concentrations ranging (40–640 mg L^−1^). An end-over-end shaker was used to equilibrate Pb-adsorbent mixtures for 24 h (predetermined equilibrium adsorption time). The Pb(II) concentrations in the supernatant solutions were determined as aforesaid in adsorption kinetics experiments. Langmuir, Freundlich, Elovich, Temkin, Fowler–Guggenheim (FG), Kiselev, and Hill-de Boer mathematical isotherm models were utilized to express the obtained sorption data. Feasibility of the adsorption process on the nanocomposite was evaluated by calculating kinetics and thermodynamics of the process, at three different temperatures (287, 297and 307 K), three pH values (4, 7, and 9), and different Pb(II) concentrations (100, 250, 500 and 1000 mg L^−1^).

To demonstrate the effect of the presence of 3 competing cations (Ni, Zn, Cu) on Pb(II) sorption, batch adsorption technique was executed with three different adsorbent doses (0.1, 0.2, and 0.3) at 500 mg L^−1^ initial Pb (II) concentration.

### Thermodynamic parameters

The thermodynamic parameters including change in the Gibbs free energy (ΔG°, J mol^−1^), enthalpy (ΔH°, J mol^−1^) and entropy (ΔS°, J mol^−1^ K^−1^) were studied to understand the effects of temperature on Cd adsorption process. These parameters were determined using the following equations:

1 $$ \Delta {\text{G}}^\circ \, = \, - {\text{RT ln K}}_{{\text{c}}} , $$

2 $$ {\text{Kc}} = {\text{ C}}_{{{\text{qe}}}} /{\text{C}}_{{\text{S}}} , $$

where R = The gas constant [8.314 kJ (mol^−1^ K^−1^)], K_c_ = the equilibrium constant, C_qe_ = The amount of Pb(II) adsorbed on the adsorbent from the solution at equilibrium (mg L^−1^), C_S_ = The equilibrium concentration of Cd(II) in the solution (mg L^−1^), The qe of the Langmuir model was used to obtain C_qe_ and C_S_.

ΔH° and ΔS° were calculated from the plot of ΔG° versus T (Fig. 8), by the following equation:

3 $$ \Delta {\text{G}}^\circ \, = \, \Delta {\text{H}}^\circ - {\text{T}}\Delta {\text{S}}^\circ . $$

### Adsorbent reusability

The economic feasibility and the reusability of (nMgO*-*Bentonite) nanocomposite were tested through regeneration of the spent nanocomposite adsorbent and use in practical applications. In general, 1 g of nanocomposite was shaken with 10 and/or 100 mg L^−1^ Pb(II) solution for 2 h using an orbital shaker. After equilibration, the Pb(II) loaded nanocomposite was dried and soaked in 50 mL HCl (0.01 M), then shaken for 2 h at room temperature. After each treatment, the adsorbent material was separated from the Pb(II) solution, washed with distilled water and finally soaked in distilled water overnight. After filtration, the solid residue was again agitated with the fresh 40 mL Pb(II) solution (10 and/or 100 mg L^−1^) for another cycle. The process was repeated for up to 6 successive cycles and the Pb(II) removal efficiency was computed after each cycle.

### The Pb(II) adsorptive removal efficiency of nanocomposite

#### Bach study

The efficiency of Pb(II) removal by nanocomposite from real wastewater through batch experiments was investigated. This real wastewater was transported from Al-Bilali agricultural drainage and industrial drainage of Rakta company for Paper manufacturing. The chemical analysis of the wastewaters used in the study is presented in Table S2. The agricultural drainage contains a low percentage of Pb (0.16 mg L^−1^), so drainage water was spiked with Pb(II) until it reached a concentration of 5 mg L^−1^.

#### Column (continuous) study

The efficiency of nanocomposite (nMgO and Bentonite) for Pb (II) removal from the industrial drainage sample that contains 1.13 mg Pb L^−1^ and the spiked agricultural drainage that contains 5 mg Pb L^−1^ was performed. The schematic diagram of the experimental design is illustrated in Fig. S1 (Supplementary Materials). Briefly, a down flow reactor filled with mixed (nMgO–Bentonite) composite and sand in PVC columns 20 cm high with 2.5-cm internal diameter were used. A peristaltic pump was continuously transporting lead-containing solution to a reservoir attached to the column. Solutions flew down through the columns and leachate were collected periodically and analyzed.

## Results and discussion

### Surface and chemical characteristics of the two adsorbents

#### Bentonite

**Figure 1:**
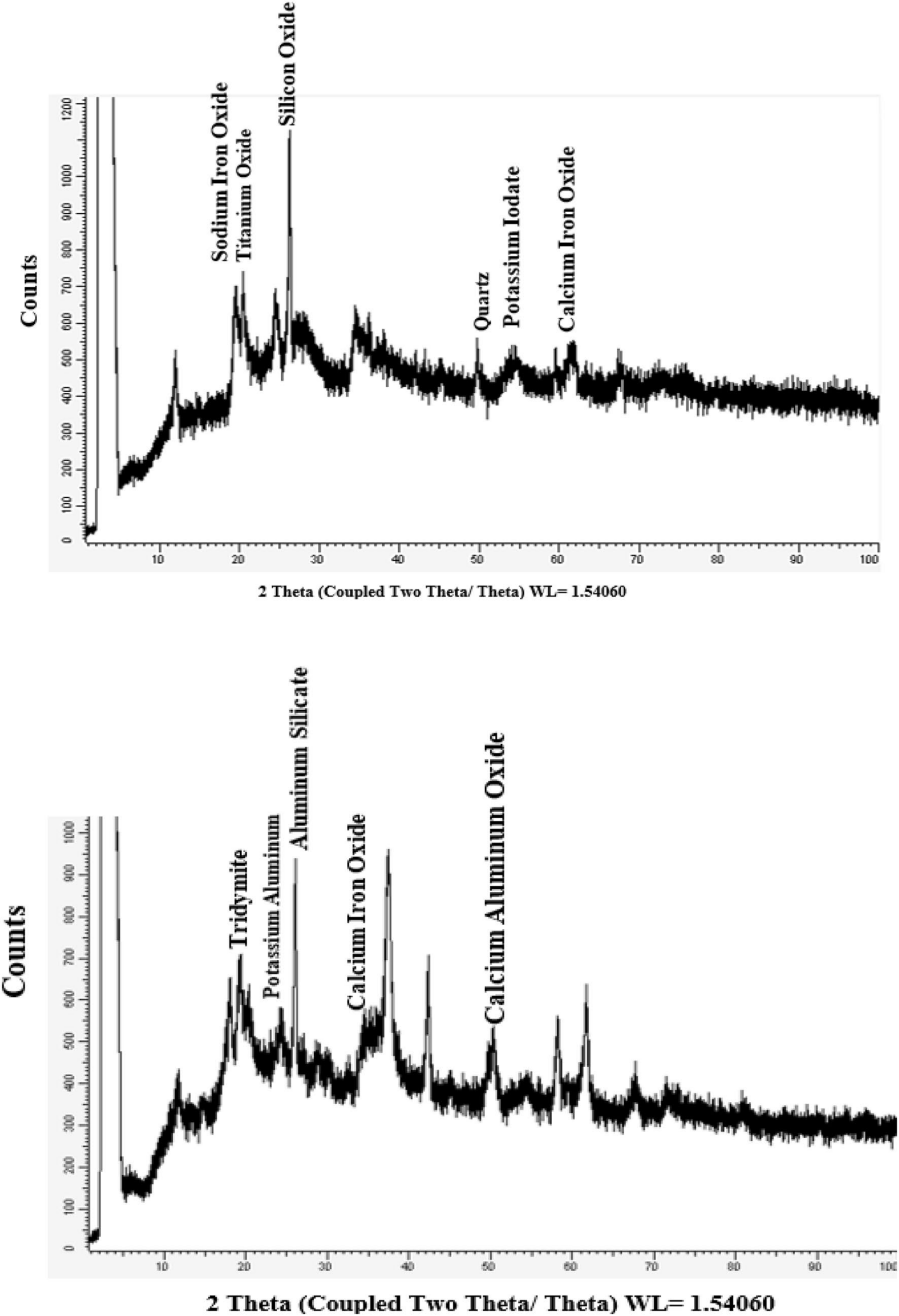
The X-ray diffraction (XRD) analyses of bentonite (above) and nano-composite (bottom).

The SEM image of bentonite sample showed that bentonite particle sizes range is within the nanometer domain range (< 100 nm). The major elements of bentonite using EDX analysis are O, Na, Mg, Al, Si and Fe in 53.08, 2.25, 1.14, 10.08 and 23.38% respectively (data not shown). The XRD of bentonite elucidated that the bentonite main contents are silicon oxide (2θ = 26.495, 28.25%), sodium iron oxide (2θ = 19.739, 14.2%), titanium Oxide (2θ = 20.41 and 11.83%), Quartz (2θ = 50.43 and 9.42%), Potassium Iodate (2θ = 54.09 and 11.25%) and calcium iron oxide (2θ = 62.26 and 8.71%) (Fig. 1 upper).

#### The nanocomposite (nMgO-bentonite)

**Figure 2:**
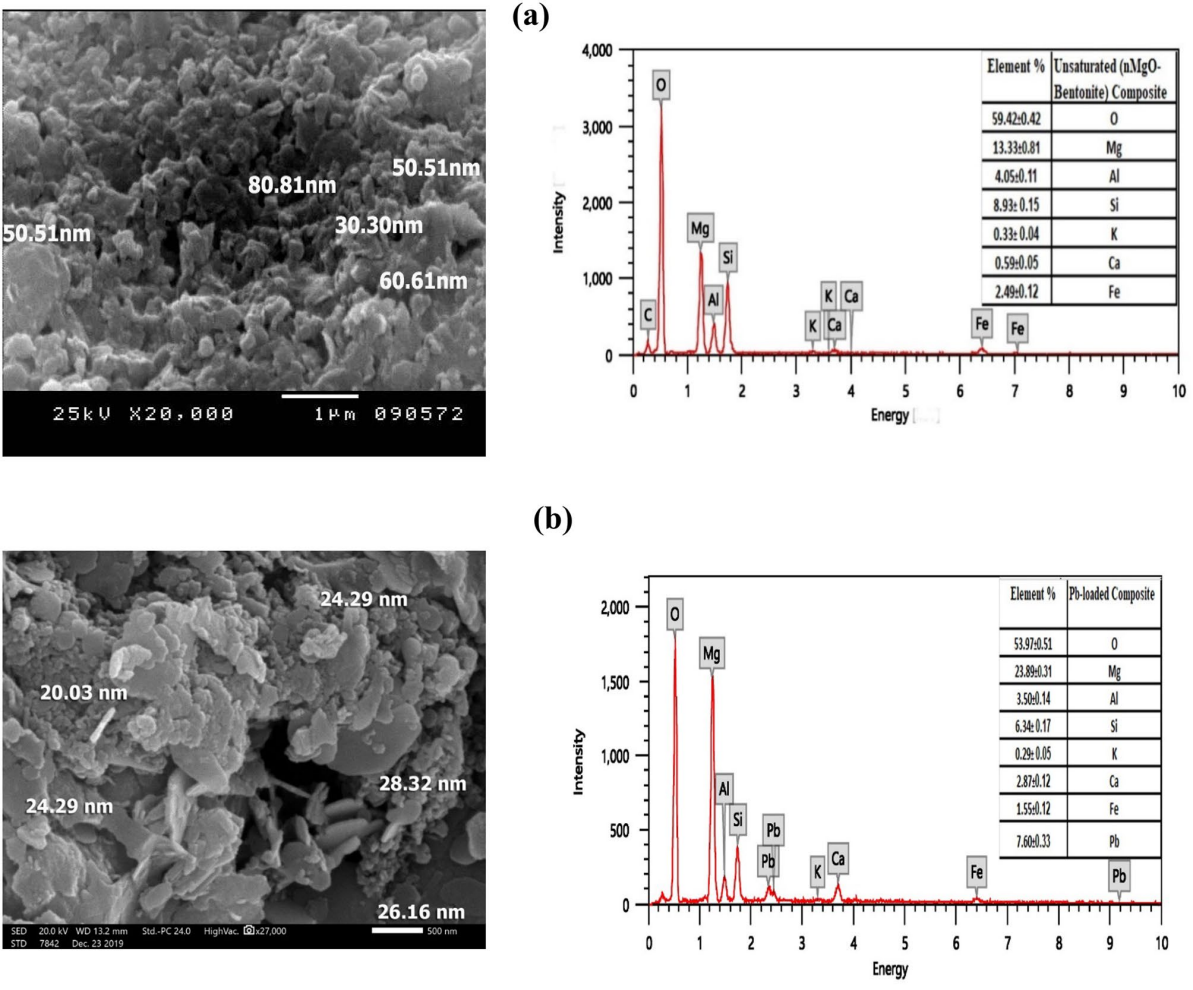
Scanning electron microscopy (SEM) image and energy-dispersive X-ray (EDX) spectrum of (**a**) nanocomposite and (**b**) Pb-saturated nanocomposite.

The SEM and EDX analyses of nanocomposite (nMgO-bentonite) are illustrated in Fig. 2. It can be seen that bentonite chips are highly apparent in SEM image and spherical shaped MgO nanoparticles are scattered on bentonite surface (Fig. 2a-left). Figure S3 clearly shows that the representative particles size dimension of nanocomposite are in the range of 1–100 nm (nanostructure), with an average size of 35 nm. The BET-specific surface area of nMgO (18.897 m^2^ g^−1^) was comparatively higher than that of Bentonite (5.477 m^2^ g^−1^). The incorporation of nMgO into Bentonite has led to considerable growth of specific surface area to 15.195 m^2^ g^−1^ for the nanocomposite (Table S3). The EDX analysis of nanocomposite samples demonstrated that O (59.42%), Mg (13.33%), Al (4.05%), Si (8.93%) and Fe (2.49%) are the main elements (Fig. 2a-right). The SEM and EDX investigation of Pb laden nanocomposite is illustrated in Fig. 2b. An appreciable amount of adsorbed Pb(II) is evident (7.60%) according to EDX analysis (Fig. 2b right). The levels of existing cations in the nanocomposite such as Si, Al, K and Fe noticeably decreased from 8.93 to 6.3%, from 4.05 to 3.50%, from 0.33 to 0.29% and from 2.49 to 1.55%, respectively as a result of Pb(II) sorption. Meanwhile, the concentration of Mg and Ca increased from 13.33 to 23.89 and from 0.59 to 2.87% respectively and O decreased from 59.42 to 53.97% of the total elements as a consequence of Pb(II) adsorption (Fig. 2b-right). The XRD patterns of the nanocomposite sample mostly include high percentage of aluminum silicate (2θ = 26.189 and 38.34%), calcium iron oxide (2θ = 34.412 and 15.30%), potassium aluminum (2θ = 24.141and 14.78%), calcium aluminum oxide (2θ = 50.375 and 12.38%) and tridymite (2θ = 20.282 and 5.06%) (Fig. 1, lower).

### Fourier transmission infrared (FTIR) spectroscopy

**Figure 3:**
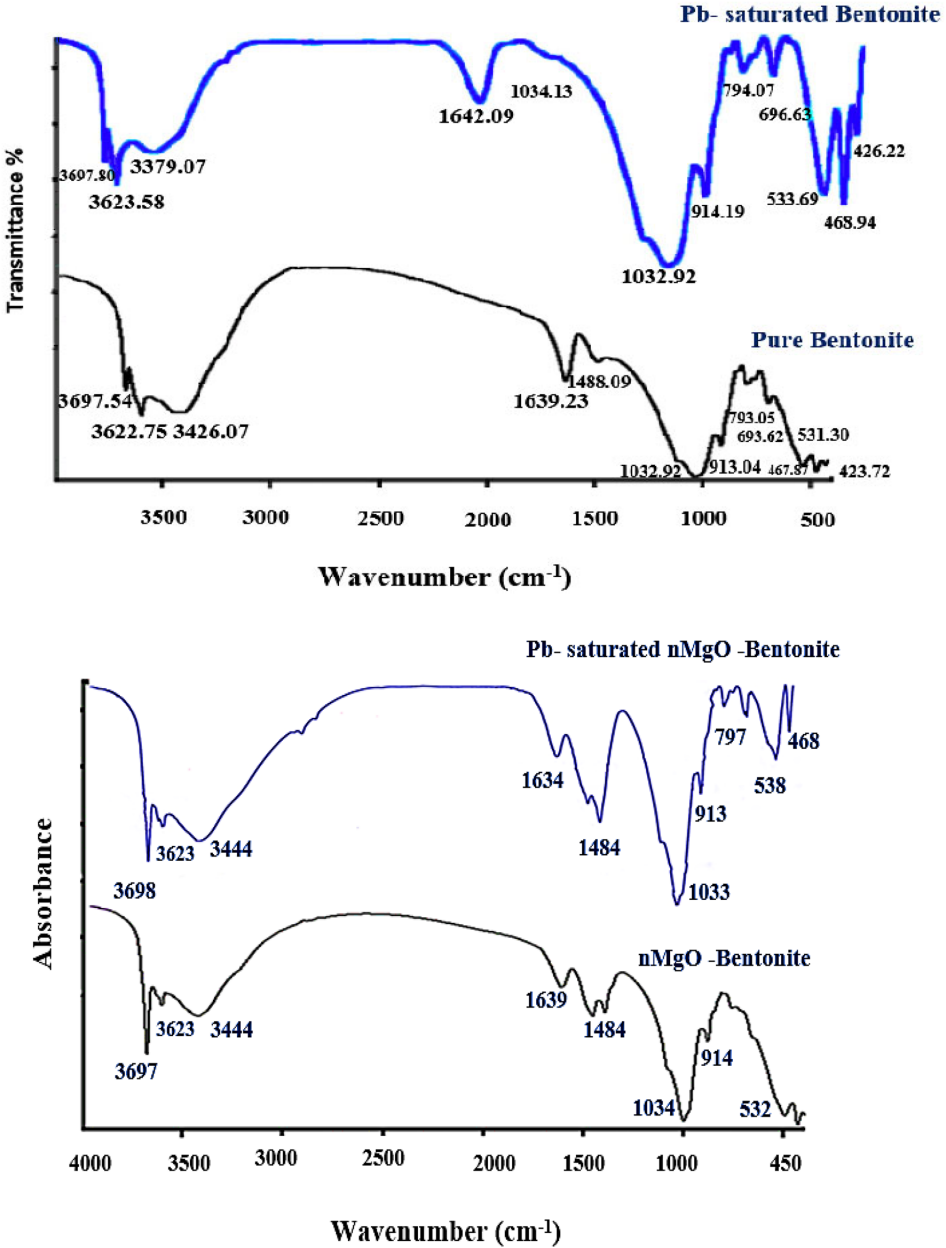
FTIR spectra of bentonite (above) and nanocomposite (bottom) before and after Pb(II) adsorption.

The spectroscopic analysis of FTIR was executed before and after Pb(II) adsorption to further explore the mechanism of Pb(II) adsorption onto bentonite and nanocomposite (Fig. 3). FTIR spectrum of bentonite before Pb(II) addition (Fig. 3 upper) shows two bands at 3697 and 3622 cm^−1^ correspond to stretching vibrations OH groups coordinated to two Al atoms. The band at 3426 cm^−1^ is assigned to OH stretching of hydroxyl groups and water present in the mineral, band at 1639 cm^−1^ is due to C=C stretching of alkene, while the two bands at 1488 and 1033 cm^−1^ are assigned to the Si–O vibration mode, and bands at 914, 534, and 468 cm^−1^ due to the vibrational modes of SiO_4_ tetrahedron^29,30^. The FTIR spectrum of Pb loaded bentonite shows disappearance of the band at 3426 cm^−1^. An intensity increase of the band at 1032 and a shift of a band at 913 cm^−1^ are observed as a consequence of the interaction with Pb(II) ions. Similarly, bands at 534 and 468 cm^−1^ are little shifted and increased in intensity due to Pb(II) ions addition. The shifts and intensity changes demonstrate the importance of OH and Si–O groups in Pb(II) adsorption by bentonite.

FTIR spectra of Pb loaded and unloaded nanocomposite (nMgO**/**bentonite) are displayed in Fig. 3, lower. The spectrum of Pb unloaded nano-composite presented two bands at 3697 cm^−1^ and 3623 cm^−1^ correspond to stretching vibrations OH groups coordinated to two Al atoms, a band at1639 cm^−1^ correspond to C=C stretching of alkene. The two bands at 1484 and 1034 cm^−1^ assigned to the Si–O vibration mode whereas the band observed at 538 cm^−1^ corresponds to the deformation mode of Al–O–Si group. Retention of Pb(II) on the surface of the nanocomposite (nMgO and Bentonite) leads to some spectral changes. The bands at 1639 cm^−1^ and 1034 cm^−1^ increased in intensity and shifted to 1633 and 1033 cm^−1^, respectively whereas bands at1484, and 538 cm^−1^ increased in intensity. Such changes indicate the active rules of Al–O–Si, Al–OH–Al, Si–O stretching groups in Pb(II) removal by nanocomposite (nMgO-bentonite)^31^.

### Adsorption kinetics and modeling

**Figure 4:**
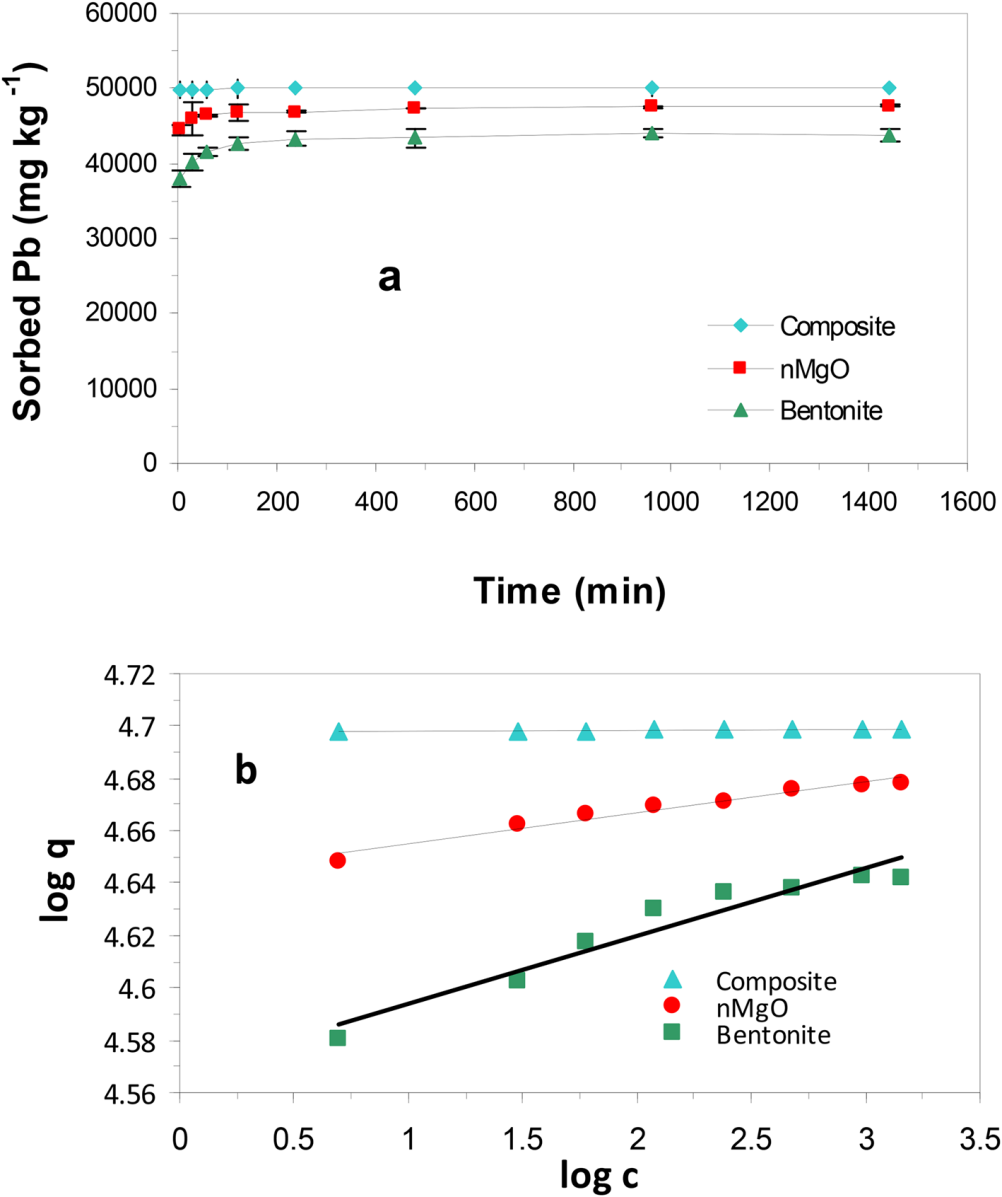
Kinetics of Pb(II) sorption by bentonite and nanocomposite (nMgO-bentonite) (**a**) and power function model for Pb sorption by the two sorbents (**b**) at Initial Pb concentration of 500 mg L^− 1^.

Adsorption kinetics were carried out to identify the exposure time needed to reach the Pb(II) adsorption equilibrium onto bentonite, and nanocomposite (Fig. 4a). Such information is crucial since time of equilibrium is the guiding principle for low-cost wastewater treatment application^32^. The adsorption kinetics of Pb(II) by the two studied adsorbents exhibited a biphasic adsorption reaction, a rapid adsorption in the initial 5 min. followed by a slow adsorption afterwards. The percentage of Pb(II) adsorbed by nanocomposite (nMgO-bentonite) at 298 K reached ~ 94% in the first five min. and followed by slow adsorption (Fig. 4a).

Kinetics adsorption data were expressed using four mathematical models for further description of Pb(II) adsorption mechanisms^33^. First-order^34^, Elovich^35^, intraparticle diffusion^36^, and modified Fruendlich^37^ kinetic models were employed. The compliance of the experimental data and the predicted model values is based on the determination coefficient (R^2^) and the standard error of estimate (SE) values. A high R^2^ and low SE values are indicators for the successful description of the model to Pb(II) adsorption kinetics. The Kinetics model constants, R^2^ and SE values for Pb(II) adsorption by Bentonite and nanocomposite (nMgO-bentonite) are given in Table 1. The R^2^ values obtained for power function model (Table 1) were higher and SE values were lower than R^2^ and SE of the other kinetic models studied.This confirmed that the adsorption process of Pb(II) by the adsorbents studied followed power function model (Fig. 4b) which suggests that chemisorption is the dominant adsorption mechanism^38^.

**Table 1:** Kinetics model constants and determination coefficients and standard error of estimate for Pb(II) adsorption by bentonite and composite sorbents.

Models	Parameter	Bentonite	Nano-composite
Elovich q_t_ = (1/β) ln(α β) + (1/β) lnt	α (mg g^−1^ min^−1^)	1.15E+20	1.25E+90
	β (mg g^−1^)	0.0010	0.0041
	R^2^	0.723	0.974
	SE	1081.751	8.198
First order ln (q_ο_ − q) = a − k_a_ t	K_d_ (min^−1^)	− 0.0082	− 0.003
	a (µg g^−1^)	8.193	4.847
	R^2^	0.794	0.955
	SE	2.365	0.163
Parabolic diffusion q = a + k_a_t^1/2^	K_d_ (µg g^−1^ min^−1/2^)	109.21	3.5773
	a (μg g^−1^)	40,501	49,865
	R^2^	0.450	0.923
	SE	1657	14.23
Power function q = *k*_*a*_ C_o_ t^1/m^	K_a_ (min^−1^)	36.98 × 10^3^	50.79 × 10^3^
	1/m	0.0233	0.0005
	R^2^	0.932	0.956
	SE	0.0012	0.0004

*q* or *q*_*t*_ = Pb adsorbed (mg kg^−1^) at time *t*, *q*_*o*_ = Pb adsorbed (mg kg^−1^) at equilibrium, *k*_*a*_ = apparent sorption rate coefficient, α = the initial adsorption rate (mg g^−1^ min^−1^), β = a constant related to the extent of surface coverage (mg g^−1^), *a* = a constant; *k*_*d*_ = apparent diffusion rate coefficient, *q* = adsorbed Pb (mg kg^−1^), *C*_*ο*_ = initial Pb concentration (mg L^−1^), *t* = reaction time (min), *k*_*a*_ = sorption rate coefficient (min^−1^), and 1/*m* = constant. *R*_2_ = determination coefficient, *SE* standard error of estimate.

### Adsorption isotherms

**Figure 5:**
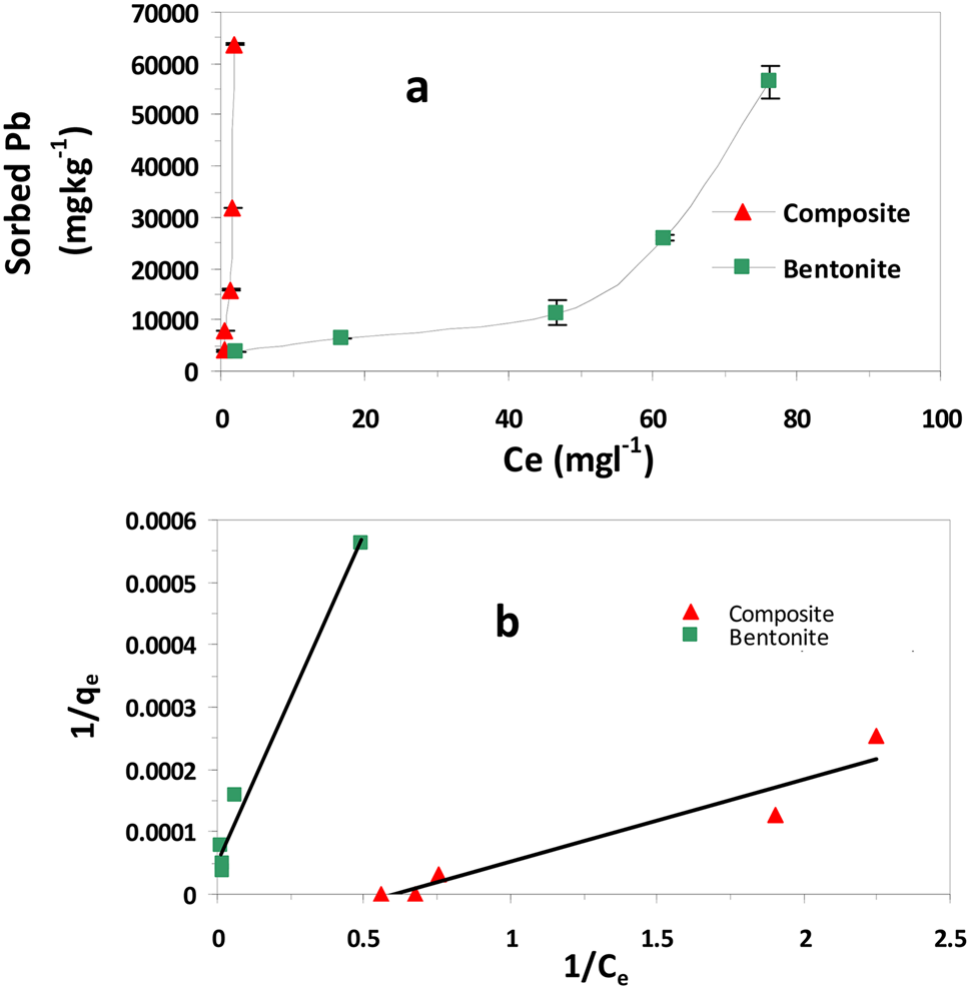
Lead adsorption isotherms for bentonite and nanocomposite (nMgO-bentonite) (**a**) and Langmuir isotherms model for the two sorbents (**b**).

Adsorption isotherms for Pb(II) were explored to estimate the sorption capacity of nMgO, bentonite and nMgO/Bentonite nanocomposite at initial concentrations range of 40–640 mg L^−1^. As shown in (Fig. 5a), the amount Pb adsorbed by the two studied adsorbents noticeably increased with the increase in initial Pb(II) concentration from 40 to 640 mg L^−1^. The amount of Pb(II) adsorbed by the nanocomposite (nMgO/bentonite) was much higher than that of bentonite as indicated by the H-type adsorption isotherm of the nanocomposite (Fig. 5a), whereas bentonite followed S-type isotherm of Giles et al.^39^. Such findings clearly showed that coating bentonite with Mg oxides nanoparticles greatly enhanced Pb adsorption capacity of bentonite adsorbent.

### Adsorption modeling

Six adsorption isotherms models were assessed for their ability to accurately predict Pb(II) adsorption by bentonite and nanocomposite. The models used were Langmuir, Freundlich, Elovich, Temkin, Fowler–Guggenheim (FG), Kiselev, and Hill-de Boer (Table 2). The models and its calculated parameters are presented in Table 2. The highest R^2^ and the smallest SE values of the studied models were the used criteria for chosen the best model capable of describing Pb(II) adsorption by bentonite and nanocomposite^40^. Lead adsorption data were best described by Langmuir model due to its highest R^2^ and Lowest SE values (Table 2, Fig. 5b) which reflects the homogeneous nature of the adsorbents surfaces. The calculated Langmuir maximum adsorption capacity (q_max_) value of nanocomposite (75 mg g^−1^) was 4.5 times higher than that of bentonite (16.66 mg g^−1^) (Table 2). Furthermore, the adsorption capacity of nMgO for Pb(II) was calculated and the q_max_ value of nMgO was found to be 55 mg g^−1^. Indeed, incorporating nMgO in the structure of the nanocomposite significantly enhanced Pb(II) removal capacity of the nanocomposite from Pb(II) contaminated water.

**Table 2:** Equilibrium model constants and determination coefficients and standard error of estimate for Pb(II) adsorption by bentonite and composite sorbents.

Models	Parameter	Bentonite	Nano-composite
Freundlich q_e_ = K_F_C_e_^1/n^	*K*_*F*_ (mL g^−1^)	1824	16,937
	1/n	0.625	1.622
	R^2^	0.742	0.891
	SE	0.635	0.419
Langmuir q_e_ = q_max_(K_L_ C_e_/1 + K_L_C_e_)	q_max_ (μg g^−1^)	16,666	75,000
	K_L_ (L mg^−1^)	1.50E−01	0.4
	R^2^	0.981	0.921
	SE	5.41E−05	3.81E−05
Elovich q_e_ /q_m_ = K_E_ C_e_exp(− q_e_/q_m_)	q_max_ (μg g^−1^) (μg g^−1^)	1,250,000	50,000
	K_E_ (L mg^−1^)	683,648,264	495,202,973
	R^2^	0.0005	0.881
	SE	0.905444	0.214333
Temkin θ = RT/∆Q lnK_0_C_e_	ΔQ (kJ mol^−1^)	4.2259	2.0009
	*K*_0_ (L g^−1^)	2.835307	2.309855
	R^2^	0.451	0.6611
	SE	1.112	0.655
Fowler–Guggenheim(FG) K_FG_C_e_ = θ/1 − θ exp(2 θ w/RT)	*W* (kJ mol^−1^)	2.581	-0.713
	*K*_FG_ (L mg^−1^)	6.563	1.684
	R^2^	0.420	0.344
	SE	0.799	0.292
Kiselev k_1_C_e_ = θ/(1 − θ) (1 + k_n_ θ)	*k*_1_ (L mg^−1^)	0.152	0.6596
	*kn*	0.88	− 1.014
	R^2^	0.844	0.483
	SE	0.126	1.898
Hill–deBoer K_1_C_e_ = θ/(1 − θ) exp(θ/ (1 − θ) − K_2_θ/RT)	K_1_ (Lmg^−1^)	16.716	0.647
	K_2_ (kJ mol^−1^)	2.21	0.249
	R^2^	0.298	0.0095
	SE	0.675	0.099

*qe* (mg g^−1^) = Pb adsorbed per gram of adsorbent, *Ce* (mg L^−1^) = equilibrium Pb concentration in solution, *K*_*F*_ = a constant related to adsorption capacity of the adsorbent (mL g^−1^), *n* = a constant, *q*_max_ (mg g^−1^) is the maximum adsorption capacity of the adsorbent, *KL* (L mg^−1^) = Langmuir constant related to the free energy of adsorption, θ = fractional coverage, *R* = the universal gas constant (kJ mol^−1^ K^−1^), *T* = the temperature (K), Δ*Q* = (− Δ*H*) the variation of adsorption energy (kJ mol^−1^), and *K*_0_ = Temkin constant (L mg^−1^), *K*_FG_ = Fowler–Guggenheim constant (L mg^−1^), *w* = the interaction energy between adsorbed molecules (kJ mol^−1^), *k*_1_ = Kiselev constant (L mg^−1^), *k*_*n*_ = a constant of complex formation between adsorbed molecules, *K*_1_ = Hill–de Boer constant (L mg^−1^), and *K*_2_ (kJ mol^−1^) = a constant related to the interaction between adsorbed molecules. A positive *K*_2_ means attraction between adsorbed species and a negative value means repulsion.

### Optimization of parameters affecting Pb removal by nanocomposite

#### Initial solution pH

**Figure 6:**
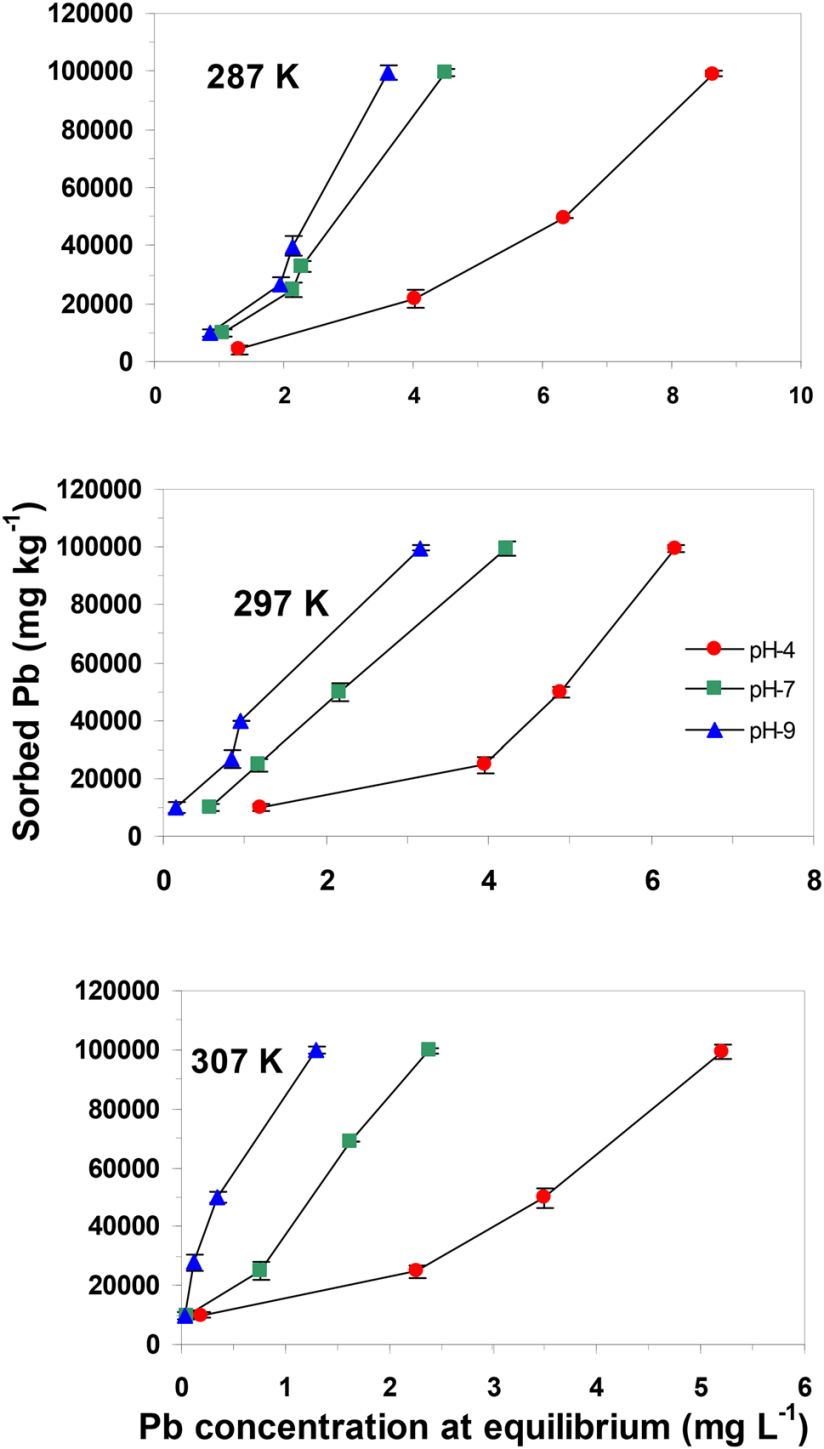
Effect of pH on Pb(II) adsorption by nanocomposite (nMgO-bentonite) at different temperatures (287, 297, and 307 K).

The influence of initial pH ranged from 4 to 9 on the adsorption process was monitored at 0.2 g adsorbent dose and temperature range of 287–307 K. Figure 6 shows that the amounts of Pb(II) ions removed was low (24,836 mg kg^−1^) at pH 4 then gradually increased until peaking to about (99,881 mg kg^−1^) at pH 9. Differences in Pb(II) ions removal with regard to pH is referred to surface properties of the adsorbent material and Pb(II) ionization state^41^. To identify the surface charge characteristics of the adsorbent, the pHpzc of nanocomposite was determined and the plot of pH_ZPC_ for the nanocomposite is depicted in Fig. S2. The results indicate that the surface of nanocomposite is highly alkaline (pH_ZPC_ = 10.9) which suggests the presence of strong alkaline groups. At pH values below pH_ZPC_, the surface of nanocomposite is positively charged while at pH above pH_ZPC_ the surface of nanocomposite is negatively charged^42^. Thus, at solution pH lower than 10.9, the nanocomposite surface possess positive charges and the negatively charged ions becomes more accessible. Meanwhile at lower pH values (pH 4–7) the surface of nanocomposite undergoes surface protonation due to the frequent interaction and accumulation of H+ ions from the bulk, which have the tendency to surround the surface of the adsorbent. Besides that, the nanocomposite surface is assumed to release the basic OH^−^ ions into the bulk which results in a slight increase in the final pH of the suspension. It is rational that at low pH(4), the (nMgO-bentonite) nanocomposite surfaces is surrounded by (H^+^) ions that compete with Pb(II) ions and lead to a low Pb(II) percentage removal. While at pH 9 the repulsive forces of positively charged metal ions and nanoparticles are reduced and Pb(II) removal accordingly increased.

#### Competitive cations

**Figure 7:**
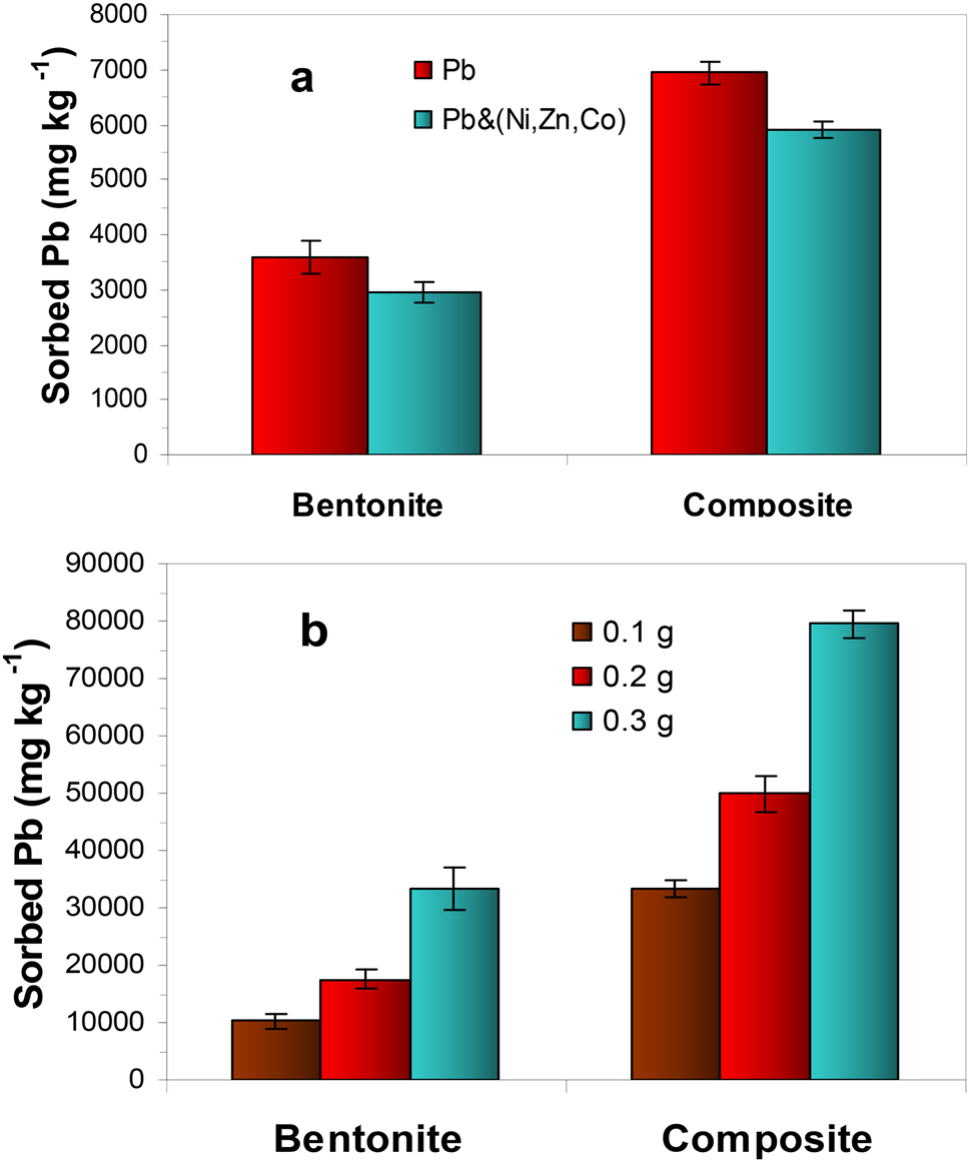
Effect of competitive cations (Ni, Zn, Cu) (**a**), and adsorbent dose (0.1, 0.2, 0.3 g) (**b**) on Pb(II) adsorption by the two sorbents.

Bach adsorption experiments were performed in the absence and presence of (Zn, Co and Ni) at concentration equal to Pb(II) concentration to study the competition effects of certain cations on the amounts of Pb(II) adsorbed by the two adsorbents studied (Fig. 7a). For instance, The amount of Pb(II) removed by bentonite from the single system was higher (3596.7 mg kg^−1^) than the amount of Pb(II) removed from combined-elements (Pb + Zn + Co + Ni) system (2951.3 mg kg^−1^). This could be referred to the competition between heavy metal ions for the binding sites available on the adsorbent surfaces and to ionic strength of the solution since the cations are charged species. These observations are in good agreement with experimentally observed results in regard to the capability of different adsorbents to bind heavy metals in the existence of competing cations^43–45^.

#### Adsorbent dose

Adsorbent capacity for a specified initial concentration depends to great extent on the adsorbent dosage. The effect of different masses (0.1–0.3 g) of nanocomposite and bentonite on Pb(II) uptake percentage was studied and the results are exhibited in Fig. 7b. The results shows that increasing the adsorbents dosage from 0.1 to 0.3 g has led to remarkable increase in the amounts of Pb(II) removed owing to the consequence increase of surface area and hence the number of available active sites responsible for Pb(II) removal (Fig. 7b).

#### Temperature

The temperature effect on the mass transfer of solutes is distinctly complex. Therefore, effect of temperature on Pb(II) adsorption on nanocomposite adsorbent was studied at three different temperatures (287, 297and 307 K) and at (100, 250, 500 and 1000) mg L^−1^ Pb(II) concentrations (Fig. 6). Increasing the reaction temperature from 287 to 307 K, with all other conditions remain constant, increased Pb(II) adsorption. The Langmuir adsorption capacity of nanocomposite for Pb(II) increased from 75 to 145 mg g^−1^ with increasing temperature from 287 to 307 K. The highest efficacy of Pb(II) removal by nano composite was at temperature 307 K and pH 9. Site^46^ pointed out that transport of metals to the reactive sites via chemisorption sorption is intensified at higher temperatures.

#### Thermodynamic parameters of Pb(II) adsorption on nanocomposite

To better comprehend the nature of Pb(II) adsorption, the thermodynamic parameters of Pb(II) adsorption by nanocomposite were calculated using the equilibrium constants under different experimental conditions^47^.

**Figure 8:**
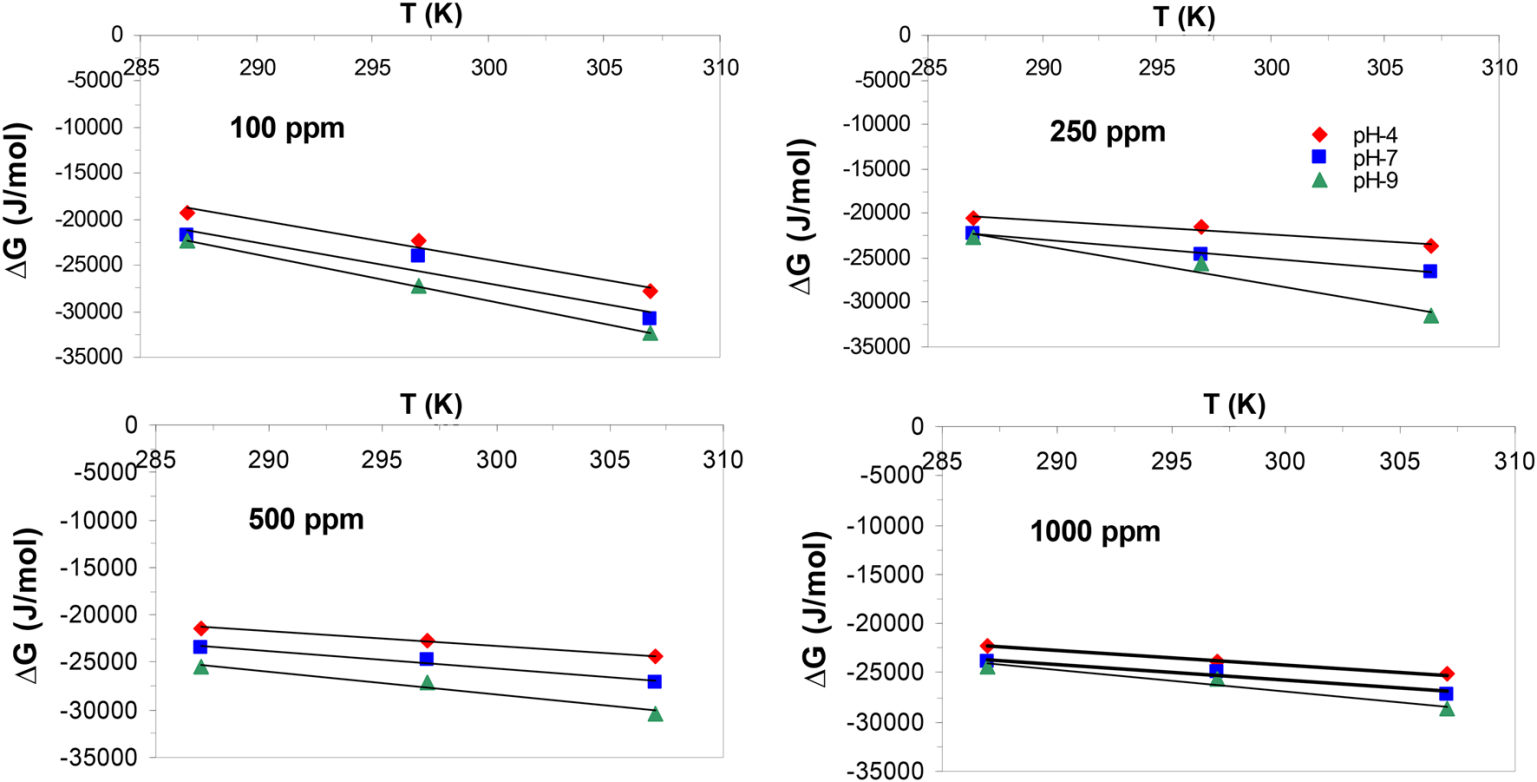
Arrhenius plot of Pb(II) adsorption on nanocomposite (T = 287, 297, and 307 K; pH 4, 7, and 9; Pb concentrations = 100, 250, 500 and 1000 mg L^−1^).

The standard free energy changes (ΔG°) for Pb (II) sorption onto nanocomposite at 287, 298 and 307 K, pH 9 and initial concentration of 100 mg L^−1^ were observed to be − 25.522, − 27, 110 and − 30.339 kJ mol^−1^, respectively (Table 3). The negative values of ΔG° indicate that Pb(II) adsorption process on nanocomposite adsorbent is feasible and spontaneous^48^. It is also noticed that the increase of ΔG◦ negative values with increasing temperature suggests the increase of the adsorption extent^49^ (Table 3, Fig. 8). In addition, it is observed a negativity increase in ΔG° values with increasing solution pH values which indicates more Pb(II) adsorption with increasing pH values from 4 to 9^50,51^.

**Table 3:** Thermodynamic parameters for Pb (II) adsorption by nanocomposite (nMgO-bentonite) sorbent at different solution pH values (4–9) and 4 initial Pb concentrations.

Initial concentration (mg L^−1^)	pH	T (K)	ΔG° (J mol^−1^)	ΔS° (Jmol^−1^ K^−1^)	ΔH° (J mol^−1^)
100	4	287	− 19,205.995	− 430.65	104,787
		297	− 22,320.261		
		307	− 27,818.95		
	7	287	− 21,835.353	− 447.06	107,208
		297	− 24,097.944		
		307	− 30,776.65		
	9	287	− 22,302	− 507.15	123,292
		297	− 27,252.438		
		307	− 32,445.086		
250	4	287	− 20,492.731	− 162.02	26,187
		297	− 21,575.787		
		307	− 23,733.21		
	7	287	− 22,326.327	− 209.94	37,866
		297	− 24,608.278		
		307	− 26,525.145		
	9	287	− 22,737.392	− 439.64	103,935
		297	− 25,643.323		
		307	− 31,530.148		
500	4	287	− 21,385.481	− 151.25	22,063
		297	− 22,778.473		
		307	− 24,410.466		
	7	287	− 23,468.592	− 185.97	30,083
		297	− 24,790.977		
		307	− 27,187.954		
	9	287	− 25,522.361	− 240.82	43,866
		297	− 27,109.837		
		307	− 30,338.694		
1000	4	287	− 22,308.407	− 142.69	18,600
		297	− 23,867.986		
		307	− 25,162.255		
	7	287	− 23,870.732	− 164.54	23,574
		297	− 24,854.853		
		307	− 27,161.592		
	9	287	− 24,395.514	− 215.76	37,851
		297	− 25,578.599		
		307	− 28,710.624

The range of ΔS° values were − 430.65 to − 507.15 and − 142.69 to − 215.76 J mol^−1^ K^−1^ for Pb(II) adsorption on nanocomposite at different pH values and initial solution concentration of 100 and 1000 mg Pb(II) L^−1^ respectively. The ΔS° negative values designate randomness decrease at the interfacial region between solid and solution with no significant shifts take place in the inner temple of the adsorbent through the adsorption^52^. The negative ΔS° values acquired in this study suggest the involvement of the dissociative mechanism in Pb(II) adsorption process. The ΔH° positive values for Pb(II) adsorption on nanocomposite at different initial solution concentration suggest the endothermic nature of Pb(II) adsorption process^53^. The high ΔH° values of the current study (18,600–123,292 kJ mol^−1^) indicate that the dominant reaction governing Pb(II) adsorption by the nanocomposite (nMgO-bentonite) is a chemosorption reaction^54^.

#### Pb(II) adsorption mechanisms

The FTIR analyses (Fig. 3 upper) revealed Pb(II) precipitation with OH^−^ groups since the intensity of the OH-peak was increased after Pb(II) adsorption onto nMgO surfaces. In addition, the appearance of the new bands at 877 cm^−1^ and 684 cm^−1^ indicates the role of carbonate in Pb(II) precipitation^55,56^. In contrast, the cation exchange reaction between Mg of nMgO and Pb(II) is excluded due to the increase of Mg percent in Pb(II) saturated nanocomposite as shown in EDX analysis (Fig. 2b-right). However, the increase of Mg% after Pb(II) adsorption highlights the role of Mg in formation of insoluble Pb(II) precipitate which merits further studies. Nevertheless, the presence of oxygen functionalized groups on nMgO may possibly attract H^+^ from aqueous solution to form OH groups and after OH ionization at alkaline conditions, Pb(II) exchange reactions are eventually taking place.The increase of the amount of Pb(II) adsorbed as the pH increases from 4 to 9 confirm such finding (Fig. 6).

Based on the above discussion, the following equations are proposed to describe the Pb(II) adsorption mechanism onto nanocomposite:

$$ {\text{MgO }} + {\text{ H}}/{\text{OH }} = {\text{ Mg}}\left( {{\text{OH}}} \right)_{{2}} , $$

$$ {\text{HO}}{-}{\text{Mg}}{-}{\text{OH}} + {\text{ Pb}}^{{{2} + }} = {\text{ HO}}{-}{\text{Mg}} - {\text{O}}{-}{\text{Pb }} + {\text{ 2H}}^{ + } \quad \left( {\text{cation exchange reaction}} \right), $$

$$ {\text{HO}}{-}{\text{Mg}} - {\text{O}}{-}{\text{Pb }} + {\text{CO}}_{{2}} + \, \left( {{\text{H}}/{\text{OH}}} \right){\text{n }} = \left( {{\text{Pb}}/{\text{Mg}}} \right)_{{3}} \left( {{\text{CO}}_{{3}} } \right)_{{2}} \left( {{\text{OH}}} \right)_{{2}} + \left( {{\text{H}}_{{2}} {\text{O}}} \right)_{{\text{n}}} \quad \left( {\text{precipitation reaction}} \right). $$

#### Reusability

**Figure 9:**
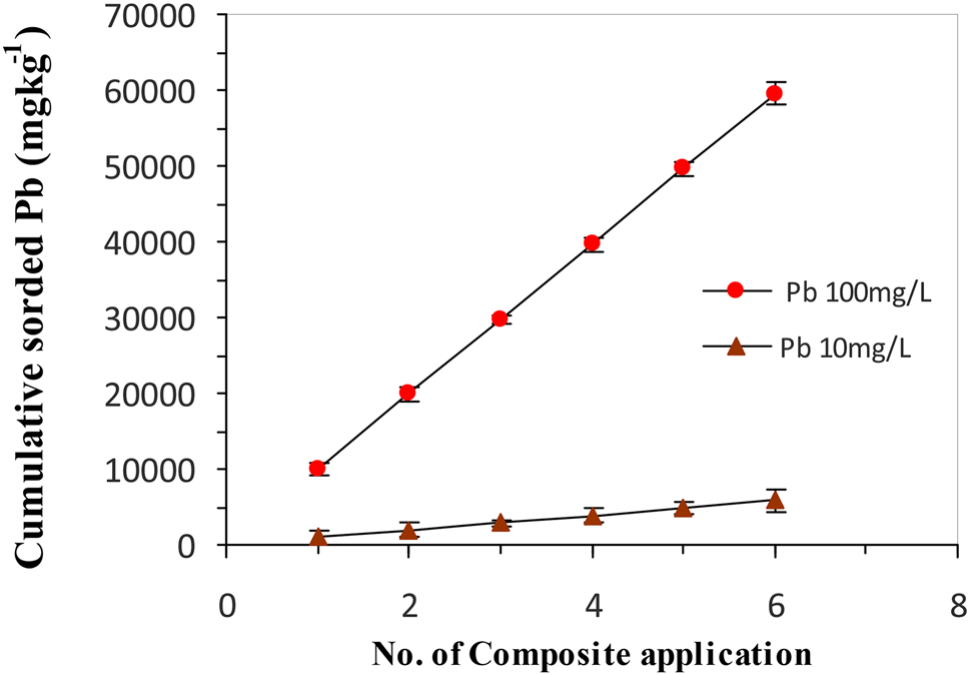
Effect of repetitive application of nanocomposite on cumulative adsorbed Pb(II) at initial Pb(II) concentrations of 10 and 100 mg L^− 1^.

From the economic and environmental points of view, the regeneration of the active sites on the spent nanocomposite adsorbent in repeated use is proportional to its stability, which is crucial for industrial applications. The paramount characteristics of an efficient adsorbent are its reusability along with significant adsorption capacity. To investigate the reusability and stability of the nanocomposite, regeneration and six adsorption–desorption successive experiments were performed at optimum status using 0.01 M HCl solution to desorb loaded Pb(II). The results showed that the amounts Pb(II) adsorbed on the nanocomposite (nMgO and Bentonite) after six successive cycles at 10 and 100 mg L^−1^ initial Pb(II) concentration didn’t significantly change manifesting the stability and the efficient reuse of the nanocomposite in the removal of Pb(II) from aqueous effluents (Fig. 9). The regeneration capability of our newly synthesized nanocomposite is of particular interest as it recovers the active sites for the Pb(II) removal from the aqueous system upon reuse. The nanocomposite has shown a consistently high Pb(II) removal efficiency (up to 90%) when used repeatedly even after 6 cycles and this reflects on its high regeneration capability and sustainability. The high adsorption efficiency of the nanocomposite highlights the practicability of the current findings and that the nanocomposite is sustainable and promising adsorbent material for Pb(II) removal from contaminated wastewater.

### The Pb(II) adsorptive removal efficiency of nanocomposite

#### Batch study

The efficiency of nanocomposite for Pb(II) removal from real wastewater through batch experiments was investigated. This real wastewater was obtained from Al-Bilali agricultural drainage and from industrial effluents of Rakta paper company (Table S2). The agricultural drainage contains a low percentage of lead (0.16 mg L^−1^). So Pb(II) solution was spiked to reach a concentration of 5 mg L^−1^. The results demonstrated the high efficiency of nanocomposite for Pb(II) removal since 94 and 96% of Pb were removed from industrial wastewater and spiked agricultural drainage, respectively.

#### Column study

The efficiency of nanocomposite (nMgO and Bentonite) for Pb(II) removal from the industrial effluents and the spiked agricultural drainage was performed. The schematic diagram of the experimental design is illustrated in Fig. S1. Briefly, a down flow reactor filled with mixed (nMgO-Bentonite) nanocomposite and sand in PVC columns 20 cm high with 2.5-cm internal diameter were used. A peristaltic pump was continuously transporting lead-containing solution to a reservoir attached with the column. Solution flew down through the columns and leachate was collected periodically and analyzed. The efficiency of nanocomposite for Pb(II) removal from industrial wastewater and drainage water were 93%, and 95%respectively. The higher Pb(II) removal efficiency from industrial wastewater compared to that of drainage wastewater was due to the complex combination of elements and contaminants in textile wastewater effluents. Overall, the findings of the current study revealed that the (nMgO–Bentonite) nanocomposite would be an outstanding ecofriendly and recycled adsorbent for efficient removal of Pb from wastewater.

#### Comparison of Pb(II) adsorption capacity onto nanocomposite with other adsorbents

The removal efficiency of Pb(II) from wastewater by the (nMgO-Bentonite) nanocomposite was evaluated by comparing its q_max_ with other adsorbents present in literature as illustrated in Table 4. Clearly, the nanocomposite showed high Pb(II)-adsorption capacity (75 mg g^−1^) as compared with other adsorbents such as Iron-coated zeolite^57^ (11.16 mg g^−1^), amidoxime-functionalized polypropylene fiber^59^ (45.64 mg g^−1^), modified corncob nanocomposite^56^ (11 mg g^−1^), magnetic calcium-rich nanocomposite^61^ (62.4 mg g^−1^) and acid functionalized magnetite nanoadsorbents^60^ (62.42 mg g^−1^). Thus, the current finding proved that (nMgO–Bentonite) nanocomposite is a promising candidate for rapid and efficient removal of lead from contaminated wastewater.

**Table 4:** Maximum adsorption capacities (q_max_) of Pb(II) adsorption onto nanocomposite and various adsorbents documented in the literature.

Adsorbent	q_max_ (mg g^−1^)	References
nMgO-bentonite nano-composite	75	Current study
Modified corncob nanocomposite	11	^56^
Iron-coated zeolite	11.16	^57^
Fe-LDH	11.51	^58^
Amidoxime-functionalized polypropylene fiber	45.64	^59^
Acid functionalized magnetite nanosorbents	62.42	^60^
Magnetic calcium-rich nanocomposite	62.4	^61^
Fe–Cu alloy coated cellulose nanocrystals	39.9	^62^

## Conclusions

The (nMgO-bentonite) nanocomposite successfully synthesized and utilized for Pb(II) removal from real wastewater. Around 94% of Pb(II) was adsorbed by nanocomposite (nMgO-bentonite) at 298 K in the first five min indicating rapid adsorption reaction. The prepared nanocomposite exhibits high Pb(II) maximum adsorption capacity (q_max_ = 75 mg g^−1^) which is 4.5-folds higher than that of bentonite. The calculated thermodynamic parameters indicate the feasibility of Pb(II) adsorption process and suggest chemisorption as the dominant reaction governing lead sorption by nanocomposite. Based on FTIR/EDX analysis, it is proposed that cation exchange and precipitation reactions are the dominant mechanisms for Pb(II) adsorption by the nanocomposite. The efficient use of the nanocomposite up to six successive adsorption–desorption cycles for adsorptive removal of Pb(II) from its aqueous solution illustrates the high capability and stability of the nanocomposite.. Application study using real wastewater through batch and column experiments showed that the efficiency of nanocomposite for Pb(II) removal from industrial wastewater and drainage water were 93%, and 95% respectively. Thus, the high efficiency of nanocomposite for Pb(II) removal from wastewater proved that nanocomposite is a promising candidate for efficient removal of Pb(II) from wastewater.

## Supplementary Information

Supplementary Information.
