# Supplementary material for: Novel metal based nanocomposite for rapid and efficient removal of lead from contaminated wastewater sorption kinetics, thermodynamics and mechanisms
Source: Sci Rep. 2022 May 19;12:8412. doi: 10.1038/s41598-022-12485-x (PMC9120188; doi:10.1038/s41598-022-12485-x)
Supplement: Supplementary file 1 — Supplementary Information. [file 41598_2022_12485_MOESM1_ESM.doc]

**Supplementary Materials**

Table (S1): Chemical characteristics of bentonite clay

| Component | Chemical composition | Content |
| --- | --- | --- |
| Silica | SiO_2_ | 56% |
| Aluminum Oxide | Al_2_O_3_ | 25% |
| Titanium Oxide | TiO2 | 1.1% |
| Iron Oxide | Fe_2_O_3_ | 7.0% |
| Calcium Oxide | Ca O | 0.7% |
| Magnesium Oxide | MgO | 3.3% |
| Potassium Oxide | K_2_O | 0.7% |
| Sodium Oxide | Na_2_O | 0.9% |
| Loss on ignition | LOI | 4.4% |

LOI, Loss on ignition

**Table S2: The chemical analysis of the wastewaters used in the study**

|  | Unit | Industrial  effluents | Agricultural drainage |
| --- | --- | --- | --- |
| EC | dSm^-1^ | 769 | 672 |
| pH |  | 9.17 | 9.39 |
| Pb | mgl^-1^ | 3.13 | 5.00 |
| Cd | mgl^-1^ | 2.73 | 5 |
| Na | mgl^-1^ | 57.14 | 5.48 |
| K | mgl^-1^ | 0.94 | 14.10 |

**Table (S3): Surface area of nMgO, bentonite and nanocomposite adsorbents**

| **Surface area (m^2^g^-1^)** | **Adsorbent** |
| --- | --- |
| **18.897** | **nMgO** |
| **5.477** | **Bentonite** |
| **15.195** | **nanocomposite** |

**
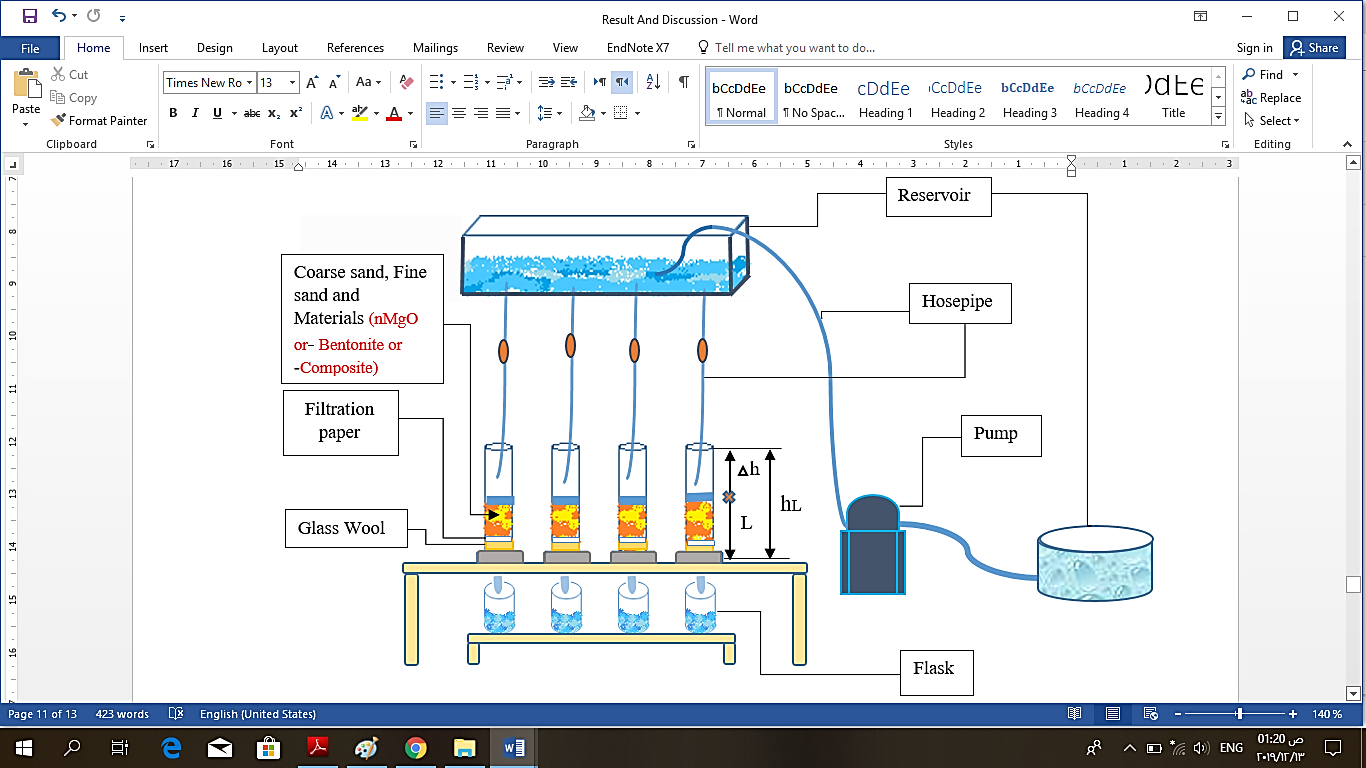
**

**Fig. (S1). The schematic diagram of the experimental column.**

**The point of zero charge (pH_PZC_)**

The pHpzc is an important feature of the adsorbents that greatly influence the adsorption process as it indicates the net surface charge of the adsorbent in solution. The pH_PZC_ of nanocompsite adsorbent was determined by using the well-known salt addition method reported in literature (Ofomaja and Ho, 2008).

**
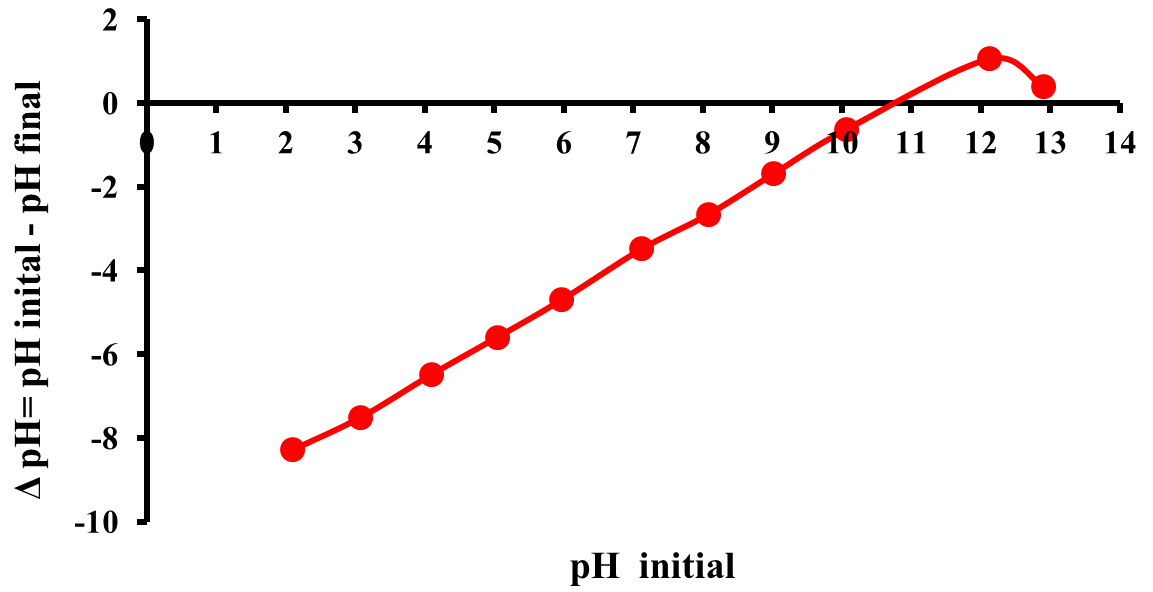
**

**Fig. (S2): The pH of point of zero charge (pHzpc) for nanocomposite adsorbent**


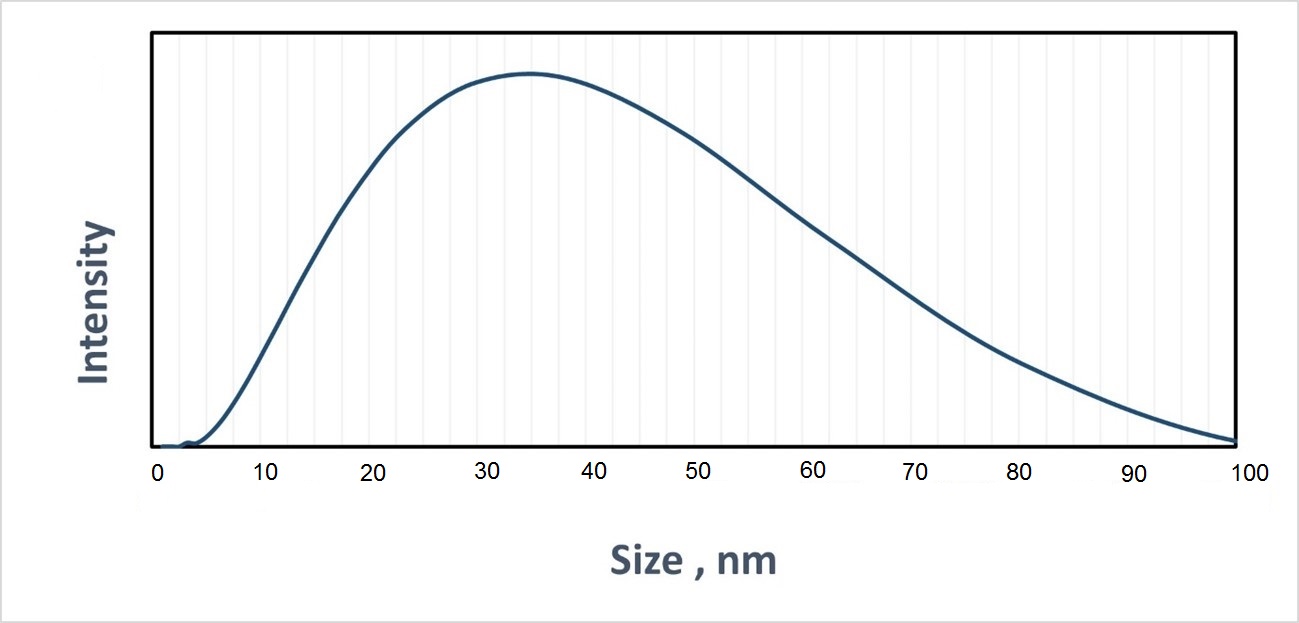


**Fig.(S3):** **Particle size distribution of nano-composite(nMgO-Bentonite)**

**References**

Ofomaja AE, Ho YS.2008. Effect of temperatures and pH on Methyl Violet biosorption by Mansonia wood sawdust. Bioresour Technol. 99: 5411–5417.
